# Plasma ApoE proteotyping for ApoE ε4 stratification in the anti-amyloid therapies era

**DOI:** 10.1186/s13195-026-02089-2

**Published:** 2026-05-21

**Authors:** Federico Coraglia, Giordano Cecchetti, Giulia Rugarli, Edoardo G. Spinelli, Alma Ghirelli, Stefano Pisano, Elisa Canu, Massimo Filippi, Federica Agosta

**Affiliations:** 1 Center for Alzheimer’s and Related Diseases (CARD), Neurology Unit, IRCCS San Raffaele Scientific Institute, Milan, Italy; 2 Neuroimaging Research Unit, Division of Neuroscience, IRCCS San Raffaele Scientific Institute, Milan, Italy; 3 Vita-Salute San Raffaele University, Milan, Italy; 4 Neurorehabilitation Unit, IRCCS San Raffaele Scientific Institute, Milan, Italy; 5 Neurotech Hub, Vita-Salute San Raffaele University, Milan, Italy

**Keywords:** Alzheimer’s disease, ApoE, Anti-amyloid therapy, Donanemab, Lecanemab, Real-world implementation

## Abstract

**Background:**

Apolipoprotein E (ApoE) ε4 status informs risk stratification and safety management for anti-amyloid therapies, but genotyping typically requires dedicated procedures, longer turnaround times, and higher costs compared with automated laboratory assays. We evaluated an automated plasma approach for ε4 stratification based on proteotyping, the quantification of isoform-specific ApoE proteins to infer ApoE genotype.

**Methods:**

We retrospectively included 110 patients (mean age 67.82 ± 10.11 years, 49.1% female) of European ancestry with cognitive impairment across a broad diagnostic spectrum, including 68 AD-spectrum cases, 29 with subjective symptoms, and 16 with other etiologies. ApoE genotyping identified 57 ε4 non-carriers (51.8%), 44 heterozygous carriers (40.0%), and 9 homozygous carriers (8.2%). ApoE4 and total ApoE (PanApoE) were measured on Lumipulse and the ApoE4/PanApoE ratio (ApoE ratio) was derived.

**Results:**

The ratio showed excellent separation across genotypes in our cohort. For ε4 carrier identification, both the ratio and ApoE4 achieved an AUC of 1.00 (95% CI 1.00–1.00), whereas PanApoE showed poor discrimination (AUC 0.30, 95% CI 0.20–0.40), reflecting lower PanApoE levels in ε4 carriers. The ratio also accurately distinguished heterozygotes from homozygotes (AUC 1.00, 95% CI 1.00–1.00).

**Conclusions:**

Automated plasma ApoE proteotyping mirrored genetic ε4 status. The ApoE ratio provided complete genotype separation, supporting its use for individual-level stratification. These findings should be interpreted cautiously considering the retrospective single-center design and the limited number of ApoE ε4 homozygotes, and require validation in larger, independent cohorts.

## Background

Alzheimer’s disease (AD) has progressively shifted from a syndromic to a biologically defined disorder, in which core pathological processes can be identified in vivo using fluid and imaging biomarkers [1]. This evolution has reshaped diagnostic frameworks and clinical reasoning, allowing AD to be conceptualized along a continuum ranging from preclinical and early symptomatic stages to dementia [1]. More recently, the advent of disease-modifying anti-amyloid therapies, particularly monoclonal antibodies targeting amyloid-β, has further accelerated this transition, shifting biomarker use from diagnosis to a key element of therapeutic decision-making in routine memory clinic practice [2–5].

Within this biological and therapeutic context, apolipoprotein E (ApoE) occupies a unique position. The ApoE gene exists in three common allelic variants (ε2, ε3, and ε4), which combine to form six possible genotypes (e.g., ε2/ε3, ε3/ε3, ε3/ε4, ε4/ε4). Individuals carrying one ε4 allele are referred to as heterozygous carriers (e.g., ε3/ε4), whereas those carrying two ε4 alleles are defined as homozygous carriers (ε4/ε4). The ε4 allele is the strongest common genetic risk factor for sporadic AD, influencing disease risk, age at onset, and multiple aspects of the biological phenotype in a dose-dependent manner [6, 7]. Importantly, ApoE ε4 status has gained direct clinical relevance in the anti-amyloid era, as ε4 carriers, particularly ε4 homozygotes, exhibit a substantially increased risk of amyloid-related imaging abnormalities (ARIA) during treatment with anti-amyloid monoclonal antibodies [2, 5, 8]. In the European regulatory context, this risk stratification has translated into concrete treatment decisions, with ε4 homozygotes currently excluded from anti-amyloid therapy by the European Medicines Agency, while ε4 heterozygotes remain eligible but require heightened monitoring [9, 10]. Accordingly, ApoE genotyping is now increasingly considered an important component of the pre-treatment work-up for anti-amyloid therapies [5, 8, 11, 12].

Despite its central role, ApoE genotyping is not optimally aligned with the demands of large-scale clinical implementation. Genetic testing requires specific consent procedures, may raise ethical and psychological considerations, and is often associated with higher costs (typically in the order of hundreds of euros) and longer turnaround times (from several days to a few months) compared to laboratory assays that can provide results within hours and at substantially lower cost (less than a hundred euros in our setting) [13–15]. In everyday clinical practice, ApoE information is frequently needed not to establish a genetic diagnosis, but to support timely therapeutic decisions. This gap highlights the need for rapid and clinically actionable ApoE ε4 stratification within routine care [14, 15].

Proteotype-based strategies, defined as approaches that infer ApoE genotype from the relative abundance of circulating ApoE protein isoforms rather than direct DNA analysis, have therefore emerged as a promising alternative. Both mass-spectrometry-based and immunoassay-based approaches have shown that plasma ApoE isoform quantification can discriminate ε4 carriers from non-carriers with high accuracy [16–18]. Ratio-based measures combining an ApoE4-specific signal with total ApoE (PanApoE) provide an internal normalization that reduces variability and emphasizes isoform composition over absolute protein levels, given that absolute plasma concentrations of ApoE isoforms may be influenced by inter-individual biological variability and pre-analytical factors.

From a translational perspective, fully automated immunoassay platforms are particularly attractive because they enable standardized, high-throughput testing within existing clinical laboratory infrastructures. The Lumipulse G System (Fujirebio), a fully automated chemiluminescent enzyme immunoassay platform, is increasingly used in clinical and research settings for cerebrospinal fluid and plasma AD biomarker analyses, making it well suited for integrating ApoE proteotyping into routine diagnostic and therapeutic workflows [19, 20]. However, data remain limited on the performance of automated plasma ApoE4 and PanApoE assays for reliably reproducing ApoE ε4 carrier status across the heterogeneous populations encountered in memory clinic settings.

In the present study, we investigated whether plasma ApoE4 and PanApoE quantification using the Lumipulse ApoE4 and PanApoE assays, and the derived ApoE4/PanApoE ratio (ApoE ratio), can support practical ApoE ε4 stratification. We evaluated in a real-world cohort genotype-dependent separation, classification performance for ε4 carrier identification, and concordance with ApoE genotyping as the reference standard.

## Methods

### Study design and setting

This was a retrospective, single‑center observational study conducted at our tertiary memory clinic (Center for Alzheimer’s and Related Diseases, CARD, Neurology Unit, IRCCS San Raffaele Scientific Institute, Milan, Italy). Consecutive patients evaluated in routine clinical practice for cognitive complaints or objective cognitive impairment were considered.

### Participants

Patients referred to our center between November 2023 and September 2025 were considered for inclusion.

All consecutive patients with available ApoE genotyping and consent for participation in plasma biomarker research studies were included.

Clinical evaluation, routine blood tests, and brain imaging (computed tomography or magnetic resonance imaging) were performed as part of standard clinical care. Additional investigations, including cerebrospinal fluid AD biomarkers, 18 F-FDG positron emission tomography, and/or DaT-SPECT, were performed in selected cases based on clinical judgment and were not required for study inclusion. Diagnostic classification was based on established clinical and biomarker-supported criteria, including NIA-AA framework for Alzheimer’s disease, and standard clinical criteria for non-AD neurodegenerative and vascular conditions, as appropriate [1, 21–25]. Cerebrospinal fluid (CSF) AD biomarkers were available in a subset of patients (*n* = 35) and were considered as supportive evidence in the diagnostic work-up when available. When CSF biomarkers were not available, plasma pTau217 (obtained in all patients) was used as an adjunctive biomarker in routine clinical practice; in the context of this study, it was considered exploratory and used only to provide additional biological and diagnostic context [26].

Blood samples for plasma ApoE proteotyping were collected in the morning under fasting conditions, at the same blood draw performed for ApoE genotyping as part of routine clinical care. Plasma analyses were conducted retrospectively for the aims of this study.

### ApoE genotyping

ApoE ε2/ε3/ε4 status was determined from genomic DNA extracted from peripheral blood collected in K2‑EDTA tubes using an automated extraction system. Genotyping targeted the two single‑nucleotide polymorphisms defining the ApoE haplotypes (rs7412 and rs429358) and was performed using an allele‑specific real‑time polymerase chain reaction (PCR) approach with TaqMan probes on a QuantStudio‑class platform (Thermo Fisher Scientific). For each sample, independent allele‑specific amplification reactions were performed alongside positive and negative controls. Based on genotyping results, participants were classified as ε4 non‑carriers, ε4 heterozygotes, or ε4 homozygotes. Genotyping was considered the reference standard for ApoE ε4 status.

### Plasma sample collection and processing

Plasma samples were obtained from peripheral venous blood collected into dipotassium EDTA tubes. Blood samples were centrifuged at 2000 g for 10 min within 3 h from collection when kept at room temperature or within 24 h if stored at 2–10 °C, according to manufacturer recommendations. Plasma was immediately aliquoted into polypropylene tubes and stored at − 80 °C until analysis. Samples underwent a single freeze–thaw cycle at the time of analysis. Storage duration varied across samples, reflecting the retrospective nature of the study, with a maximum storage time of up to approximately 2 years.

Prior to measurement, samples were thawed at room temperature for at least 30 min, vortexed briefly, and centrifuged again at 2000 g for 5 min, in accordance with manufacturer recommendations. Blood sampling was performed as part of routine clinical procedures in the morning under fasting conditions; temporal alignment with other diagnostic investigations was not required. Samples obtained for research purposes were processed and stored by the institutional biobank Biological Resource Center (CRB-OSR) (Num ID CRB in BBMRI-ERIC: bbmri-eric: ID: IT_1383758011993577).

### Plasma ApoE quantification

Plasma ApoE4 and PanApoE concentrations were measured using fully automated chemiluminescent enzyme immunoassays on the Lumipulse G System (Fujirebio). The assays employ a two‑step immunoreaction in which ApoE4 or PanApoE in the sample binds to specific monoclonal antibodies, followed by detection through an enzyme‑mediated chemiluminescent reaction, with signal intensity proportional to analyte concentration.

The analytical and diagnostic performance of the Lumipulse ApoE4 and PanApoE immunoassays has been previously evaluated in dedicated validation studies [27]. These assays are based on a fully automated chemiluminescent enzyme immunoassay (CLEIA) platform, enabling standardized and high-throughput measurements with minimal operator-dependent variability.

Calibrators and internal quality controls provided by the manufacturer were used according to standard laboratory procedures [27, 28]. Plasma ApoE4 and PanApoE measurements were performed on the Lumipulse platform across multiple analytical runs on different days using the same batch of reagents, reflecting routine laboratory conditions. The use of a fully automated platform with standardized reagent handling, calibration procedures, and controlled assay conditions ensures robust performance and supports inter-run consistency.

The ApoE4/PanApoE ratio was calculated as the proportion of ApoE4 relative to PanApoE and expressed as a percentage to facilitate interpretability and align with predefined manufacturer thresholds used for genotype classification (< 5% for non-carriers, 5–75% for heterozygous carriers, and ≥ 75% for homozygous carriers), derived and previously reported in studies using the same assay platform [27].

### Statistical analysis

Statistical analyses were designed to address two complementary aims: (i) to assess genotype-dependent differences and whether plasma ApoE measures varied according to ε4 allele count (non-carriers, heterozygotes, and homozygotes), and (ii) to evaluate the ability of plasma ApoE measures to classify ε4 carrier status at the individual level. All analyses were performed using R (version 4.5.2).

Between-group differences across ApoE genotypes (ε4 non-carriers, ε4 heterozygotes and ε4 homozygotes) were assessed using non-parametric tests (two-sided), including the Kruskal–Wallis test, followed by pairwise comparisons when appropriate. Potential confounders such as age and sex were not included as covariates, as demographic characteristics were comparable across genotype groups in the study cohort and given the exploratory nature of the analyses. In addition, previous studies using similar assay approaches have shown that ApoE isoform levels are primarily driven by ApoE genotype, with limited influence of demographic factors [27].

The discriminative performance of the ApoE ratio and plasma ApoE4 concentration for identifying ApoE ε4 carrier status (presence of at least one ε4 allele) was evaluated using receiver operating characteristic (ROC) curve analyses. Areas under the curve (AUCs) with 95% confidence intervals were calculated. Pairwise comparisons between ROC curves were performed using the non-parametric DeLong method for correlated AUCs. No missing data were present for the variables included in the analyses. No formal correction for multiple comparisons was applied, as the main analyses were pre-specified and focused on a limited number of primary outcomes. Exploratory analyses were interpreted descriptively. Given the retrospective design, no formal sample size calculation or power analysis was conducted; the sample size was determined by the number of consecutive eligible patients available during the study period.

## Results

### Study population

A total of 110 patients of European ancestry evaluated for cognitive disturbances with available ApoE genotyping were included in the present analysis. Fifty-seven patients (51.8%) were ε4 non-carriers, either ε3/ε3 or ε2/ε3, 44 (40.0%) were ε4 heterozygotes, and 9 (8.2%) were ε4 homozygotes. The distribution of ApoE genotypes was as follows: ε2/ε3 in 4 patients (3.6%), ε2/ε4 in 3 (2.7%), ε3/ε3 in 53 (48.2%), ε3/ε4 in 41 (37.3%), and ε4/ε4 in 9 (8.2%). Overall, 53 individuals (48.2%) carried at least one ε4 allele. Main demographic features of ApoE groups are detailed in Table 1.

**Table 1:** Plasma PanApoE, ApoE4, and ApoE ratio according to ApoE genotype

	ApoE ε4 non-carriers	ApoE ε4 heterozygotes	ApoE ε4 homozygotes	*p* value
*N*	57	44	9	
Age *(y)*	71.09 (61.46, 75.81) [46.15–85.60]	69.86 (56.74, 76.79) [49.77–84.03]	64.99 (56.70, 68.73) [47.73–82.14]	0.455
Sex *(F | M*,* %F)*	32 | 25 56.1%	20 | 24 45.5%	2 | 7 22.2%	0.138
ApoE4 *(µg/mL)*	0.00 (0.00, 0.00) [0.00–0.10]	3.15 (1.98, 5.06) [0.90–23.50]	21.10 (14.40, 29.50) [9.7–34.10]	< 0.001
PanApoE *(µg/mL)*	16.80 (10.25, 25.35) [2.60–49.80]	9.05 (6.63, 14.28) [2.40–38.70]	10.30 (6.60, 15.90) [4.60–20.10]	0.002
ApoE ratio *(%)*	0.00 (0.00, 0.00) [0.00–1.00]	34.20 (26.30, 41.10) [8.10–63.00]	185.50 (169.70, 210.90) [151.50–250.00]	< 0.001

Values are reported as median (Q1, Q3), and range. Continuous variables were compared across groups using the Kruskal–Wallis test, while categorical variables were compared using Pearson’s Chi-Square test

Abbreviations: *ApoE* apolipoprotein E, *ε4* apolipoprotein E epsilon 4 allele, *PanApoE* total apolipoprotein E, *ApoE4* apolipoprotein E4 isoform, *ApoE ratio* ApoE4-to-PanApoE ratio, *F* female, *IQR* interquartile range, *M* male, *ns* not significant, *Q1* first quartile, *Q3* third quartile, *y* years, *µg/mL* micrograms per milliliter

Significance: ***, < 0.001

The cohort encompassed a broad diagnostic spectrum, predominantly within the AD continuum. Specifically, 68 patients (61.8%) were classified as belonging to the AD continuum, including individuals with AD dementia (*n* = 19), mild cognitive impairment due to AD (AD-MCI; *n* = 40), and atypical AD phenotypes supported by biological evidence (*n* = 9). Twenty-nine individuals (26.4%) were presenting with subjective cognitive decline; among them, only one showed evidence of AD-related pathology. The remaining 13 patients (11.8%) had non-AD or alternative etiologies, including vascular dementia, Lewy body dementia, limbic-predominant age-related TDP-43 encephalopathy, cerebral amyloid angiopathy, psychiatric-related cognitive impairment.

### Demographic comparability across ApoE genotypes

Age and sex distribution did not differ significantly across ApoE genotype groups (Table 1). Median age was comparable among ε4 non-carriers, heterozygotes, and homozygotes (median age 71.1, 69.9, and 65.0 years for ε4 non-carriers, heterozygotes, and homozygotes, respectively; Kruskal–Wallis *p* = 0.455), with partially overlapping age ranges across groups. Similarly, the proportion of females did not differ significantly according to ε4 carrier status (56.1%, 45.5%, and 22.2% respectively; Pearson’s Chi-Square *p* = 0.138).

### Plasma ApoE4, PanApoE, and ApoE ratio across genotypes

**Fig. 1:**
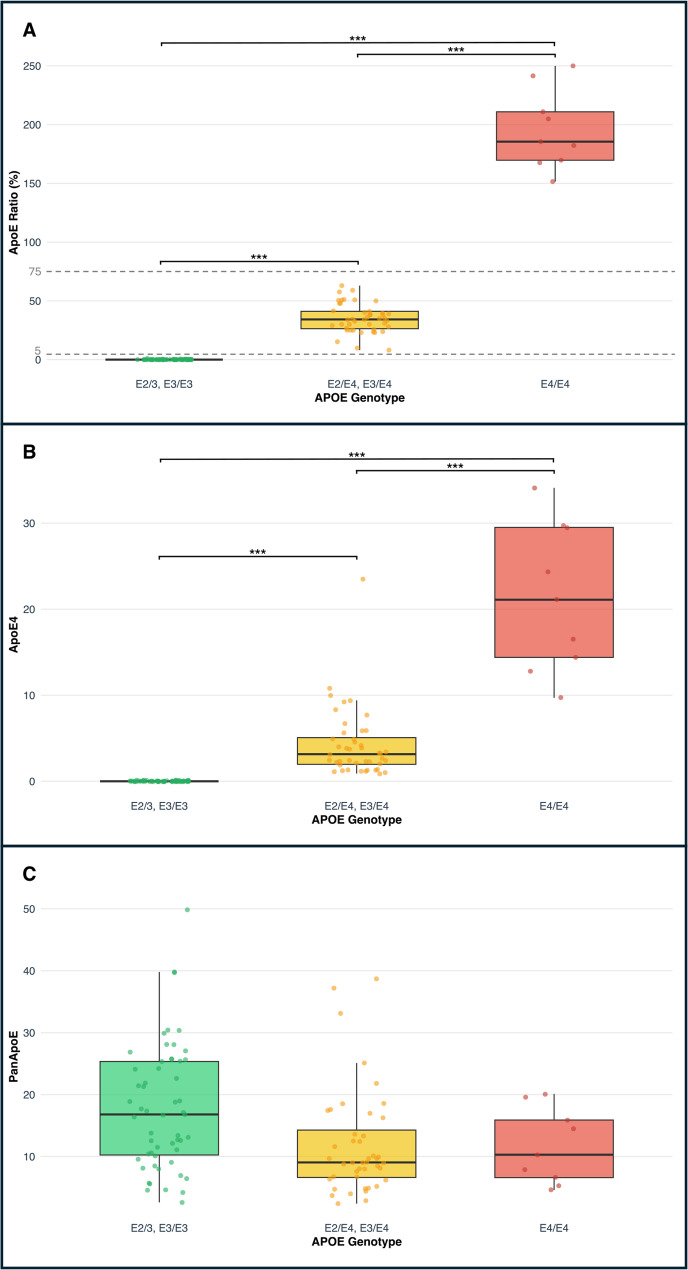
Plasma ApoE measures by ApoE genotype. Distribution of plasma ApoE-related measures across ApoE ε4 non-carriers (ε2/ε2, ε2/ε3), ε4 heterozygotes (ε2/ε4, ε3/ε4), and ε4 homozygotes (ε4/ε4). **A** Plasma ApoE ratio, dashed horizontal lines indicate manufacturer-defined cut-offs for ApoE4/PanApoE ratio (< 5%, 5–75%, ≥ 75%); **B** plasma ApoE4 concentration; **C** plasma PanApoE concentration. Boxes represent the median and interquartile range (IQR); whiskers indicate the full data range, with individual data points shown

Plasma concentrations of ApoE4, PanApoE, and the derived ApoE ratio differed across ApoE genotypes (Table 1; Fig. 1).

PanApoE levels showed a significant overall group effect (*p* = 0.002), with lower values observed in ε4 carriers compared with non-carriers (Fig. 1C). Post-hoc comparisons indicated that this difference was driven by the contrast between ε4 non-carriers (ε2/ε3, ε3/ε3), and carriers (ε2/ε4, ε3/ε4 and ε4/ε4; *p* < 0.001), whereas no significant differences were observed between heterozygous (ε2/ε4, ε3/ε4) and homozygous (ε4/ε4) carriers (*p* = 0.371). Accordingly, PanApoE distributions largely overlapped across genotype groups and did not exhibit a clear allele-dose pattern.

By contrast, both plasma ApoE4 concentration and the ApoE ratio demonstrated a clear allele-dose–dependent increase.

The ApoE ratio showed distinct genotype-dependent distributions with a highly significant overall group effect (*p* < 0.001) and showed separation across genotype groups in this cohort, with no observed overlap between ε4 non-carriers, heterozygotes, and homozygotes (Fig. 1A).

Although plasma ApoE4 concentration alone also increased markedly with ε4 allele count (*p* < 0.001), its distributions showed partial overlap between genotype groups, particularly between ε4 non-carriers and heterozygotes, but also between the heterozygous and homozygous groups, and greater inter-individual variability compared with the ratio (Fig. 1B).

### Classification performance of plasma ApoE measures

ROC analyses were conducted to assess the ability of plasma ApoE measures to identify ApoE ε4 carrier status, defined as the presence of at least one ε4 allele. Both the ApoE ratio and absolute plasma ApoE4 concentration achieved perfect discrimination between ε4 carriers and non-carriers (AUC 1.00 for both in this cohort, 95% CI 1.00–1.00), with no difference between them (ΔAUC 0.00, *p* = 1.00). In contrast, PanApoE concentration alone showed poor discrimination (AUC 0.30, 95% CI 0.20–0.40). Both ApoE4 and the ApoE ratio were significantly superior to PanApoE (ΔAUC − 0.70 for both comparisons, *p* < 0.001).

In a secondary ROC analysis focusing on genotype stratification among ε4 carriers (heterozygotes vs. homozygotes), both ApoE4 concentration (AUC 0.99, 95% CI 0.98–1.00) and the ApoE ratio (AUC 1.00, 95% CI 1.00–1.00) showed excellent discriminative performance, although this finding should be interpreted cautiously given the limited number of homozygous individuals. Both markers were significantly superior to PanApoE (AUC 0.41; ΔAUC − 0.58 and − 0.59, respectively; *p* < 0.001), with no significant difference between ApoE4 and the ApoE ratio (ΔAUC − 0.008, *p* = 0.24).

## Discussion

In this real-world memory clinic cohort spanning the AD continuum and alternative etiologies, plasma-based ApoE proteotyping derived from the Lumipulse immunoassay closely mirrored ApoE ε4 carrier status as determined by genotyping. Three distinct analytical patterns emerged: (1) a quantitative shift without genotype discrimination (PanApoE), (2) high statistical discrimination with partial overlap (ApoE4), and (3) complete genotype stratification through ratio-based normalization (ApoE ratio).

Most notably, the ApoE ratio demonstrated excellent genotype separation in our cohort between ε4 non-carriers, heterozygotes, and homozygotes, consistent with previous evaluations of Lumipulse-based ApoE proteotyping reporting high concordance with ApoE genotyping and accurate discrimination between ε4 non-carriers, heterozygotes, and homozygotes using predefined ratio cut-offs [27, 29], as well as similar patterns of genotype separation in independent clinical cohorts [30]. However, this observation should be interpreted with caution given the sample size, particularly the limited number of ε4 homozygotes, and requires confirmation in larger independent cohorts.

Importantly, age and sex distributions were comparable across genotype groups, which may reduce the likelihood that the observed differences in plasma ApoE measures were driven by major demographic confounding. However, this does not exclude the potential influence of other unmeasured confounders, such as differences in disease status or comorbidities, which were not explicitly accounted for in the analysis.

Lower plasma PanApoE levels in ε4 carriers are consistent with previous studies showing genotype-dependent differences in ApoE concentrations [31, 32]. These differences likely reflect isoform-specific biological properties, including differential turnover rates of ApoE isoforms, with ApoE4 potentially exhibiting faster plasma clearance. Moreover, the unequal contribution of ApoE isoforms to PanApoE levels further supports the biological relevance of ApoE quantification beyond genotyping. In this context, the ApoE4/PanApoE ratio may capture these underlying biological differences, translating genotype-dependent molecular features into a measurable plasma phenotype.

In contrast, although PanApoE showed a significant overall difference across genotype groups, this effect was driven by lower PanApoE levels in ε4 carriers compared with non-carriers, consistent with previous reports indicating reduced plasma ApoE concentrations in ε4 carriers [17, 18]. However, PanApoE did not demonstrate an allele-dose pattern and failed to distinguish between heterozygous and homozygous ε4 carriers, with substantial overlap between groups. These findings reinforce the notion that PanApoE reflects a genotype-associated quantitative shift at the population level, but lacks the isoform specificity required for individual-level genotype inference.

Absolute plasma ApoE4 concentration represented an intermediate pattern. From a statistical perspective, ROC analyses indicated near-perfect performance for identifying ε4 carriers, consistent with previously reported data. Nevertheless, its distributions exhibited partial overlap between heterozygotes and homozygotes. While this overlap did not meaningfully affect carrier versus non-carrier classification at the group level, it becomes clinically relevant when precise discrimination between heterozygous and homozygous ε4 carriers is required.

This distinction acquires particular importance in the evolving therapeutic landscape of AD. In the era of disease-modifying anti-amyloid therapies, ApoE ε4 status has become directly actionable, as ε4 carriers, particularly ε4 homozygotes, exhibit a substantially increased risk of ARIAs [2, 33]. Thus, ε4 stratification is not merely prognostic but directly informs treatment eligibility, monitoring intensity, and individualized risk–benefit discussions. In this framework, even limited overlap in protein distributions may have asymmetric and clinically meaningful consequences: a falsely classified homozygote (in reality heterozygous) might be inappropriately excluded from therapy and deprived of potential benefit; conversely, a falsely classified heterozygote (homozygous) might be exposed to treatment despite a markedly increased ARIA risk.

Against this background, the clear separation observed in this cohort with the ApoE ratio assumes particular relevance. Conceptually, internal normalization of ApoE4 to PanApoE enhances robustness by emphasizing isoform composition rather than absolute protein abundance. In our cohort, cases with overlapping ApoE4 concentrations across genotype groups were effectively resolved by the ApoE ratio, supporting its added value for individual-level discrimination.

Importantly, predefined manufacturer thresholds (< 5% for absence of ε4, 5–74% for heterozygosity, ≥ 75% for homozygosity), pre-specified based on previous studies using the same assay platform [27], effectively stratified patients in our cohort without the need for cohort-specific optimization, aligning with genotyping results and supporting feasibility within routine laboratory workflows. However, the observation of AUC values of 1.00 should be interpreted with caution, as these results may reflect cohort-specific characteristics and the relatively limited sample size. These findings should therefore be considered exploratory and require validation in larger, independent cohorts.

It is nevertheless important to acknowledge that rare discrepancies between genotype and proteotype have been described, including cases related to uncommon genetic variants affecting ApoE expression or detectability [34]. Although such events appear infrequent in large series with overall concordance rates exceeding 99% [29], they underscore the need for awareness of potential edge cases and for predefined reflex strategies when results are unexpected or borderline. From a biological perspective, these discrepancies may reflect situations in which proteotype-based measures capture the functional expression of ApoE isoforms, which may be more directly relevant than genotype alone. For instance, rare ApoE variants may affect protein expression, stability, or detectability, resulting in a mismatch between the expected genotype and the observed protein profile. In this context, proteotype-based stratification could provide a more functionally relevant estimate of ApoE-related biological effects in selected individuals. However, this hypothesis remains speculative and requires further investigation. From a clinical implementation perspective, such scenarios may warrant confirmatory ApoE genotyping, particularly when proteotype results are inconsistent with clinical expectations or fall close to classification thresholds.

The relatively small number of ε4 homozygotes in our cohort (*n* = 9), which reflects the relatively low prevalence of this genotype in clinical populations [35], represents a limitation and may reduce the robustness of subgroup analyses, particularly for genotype stratification.

Taken together, these findings suggest the potential feasibility a pragmatic stepwise implementation strategy. Plasma ApoE proteotyping, particularly through the ApoE ratio, may represent a promising approach as an initial screening or stratification tool in memory clinic settings, allowing rapid and laboratory-based identification of individuals likely to carry the ε4 allele. From a broader perspective, protein-based approaches may also help mitigate some of the ethical and psychological considerations associated with genetic testing, as they rely on biomarker measurements rather than direct genetic disclosure. Confirmatory genotyping could then be reserved for selected cases, such as borderline or unexpected classifications, or in contexts where regulatory or therapeutic decisions mandate genetic confirmation. However, this approach remains exploratory and requires validation in larger, independent cohorts, and cannot currently replace genotyping as the reference standard. In line with previous proposals of a stepwise approach [36, 37], such a model may balance analytical performance with clinical safety while facilitating timely ε4 stratification in the era of anti-amyloid therapies.

## Limitations

Several limitations should also be considered.

First, this was a retrospective, single-center study conducted in a tertiary memory clinic setting, and replication in independent cohorts is required to confirm generalizability across laboratories and clinical populations.

Second, the number of ε4 homozygotes was relatively small; although complete genotype separation was observed for the ratio in this dataset, larger samples will be necessary to confirm stability of discrimination, particularly at the distribution extremes.

Third, formal analytical validation and inter-laboratory harmonization studies will be essential before plasma ApoE proteotyping can be considered a substitute for genotyping within structured clinical pathways. Although all measurements in the present study were performed using the same reagent batch, additional studies assessing inter-batch variability are required to confirm analytical robustness across different laboratory conditions.

Finally, while no misclassifications were observed in this cohort, the potential impact of rare genetic variants or unexpected biological patterns warrants cautious interpretation and supports the need for predefined confirmatory strategies.

## Conclusions

Plasma ApoE proteotyping based on the ApoE ratio demonstrated excellent agreement with ApoE genotyping for identifying ε4 carrier status in a real-world memory clinic cohort.

This approach may provide a rapid and accessible laboratory-based strategy for ApoE ε4 stratification, which may be particularly relevant in the context of emerging anti-amyloid therapies requiring genetic risk assessment. However, these findings should be interpreted as preliminary and require validation in larger, independent cohorts. Although further multicenter validation is needed, plasma-based ApoE proteotyping may represent a practical first-line tool to guide patient selection for confirmatory genotyping in clinical practice.

## Data Availability

The datasets generated and/or analysed during the current study are available from the corresponding author on reasonable request.

## Abbreviations

AD: Alzheimer’s disease
ApoE: Apolipoprotein E
AUC: Area under the curve
CI: Confidence interval
CSF: Cerebrospinal fluid
PanApoE: Total apolipoprotein E
ROC: Receiver operating characteristic
